# Impact of climate-driven changes in temperature on stomatal anatomy and physiology

**DOI:** 10.1098/rstb.2024.0244

**Published:** 2025-05-29

**Authors:** Tanja A. Hofmann, William Atkinson, Mengjie Fan, Andrew J. Simkin, Pratham Jindal, Tracy Lawson

**Affiliations:** ^1^ School of Life Sciences, University of Essex, Wivenhoe Park, Colchester, UK; ^2^ Department of Plant Biology, University of Illinois at Urbana-Champaign, Urbana, IL, USA

**Keywords:** stomata, climate change, heat, stomatal density, water-use efficiency

## Abstract

Climate-driven changes in temperature are likely to have major implications for plant performance including impacts on stomatal conductance (*g*_s_), gaseous exchange, photosynthesis, leaf temperature and plant water use. Stomatal conductance is not only vital for carbon assimilation but also plays a key role in maintaining optimum leaf temperatures for cellular processes. Higher *g*_s_ facilitates both CO_2_ uptake and enhanced evaporative leaf cooling, however, most likely at the cost of greater water loss and lower water-use efficiency. Lower *g*_s_ helps to maintain overall plant water status but at the expense of C uptake with reduced evaporative cooling which, at elevated temperatures, could be lethal. It is therefore important for *g*_s_ to balance these competing demands; however, with rapid changes in temperature due to climate change, stomatal engineering may be required to ensure that this balance is achieved and different strategies for different crops in different environments may be needed. Here, we review current knowledge of stomatal anatomical and behavioural responses to temperature-driven changes, focusing on both rising temperatures and extreme heat events and potential genetic targets for manipulation of relevant stomatal characteristics.

This article is part of the theme issue ‘Crops under stress: can we mitigate the impacts of climate change on agriculture and launch the ‘Resilience Revolution’?’.

## 1. Introduction

Stomata are small adjustable pores found on most aerial parts of plants, including leaves, stems and reproductive structures [1], with each stoma consisting of two specialized cells in the epidermis (guard cells, GC) that are responsible for controlling stomatal aperture [2]. Since the leaf cuticle is largely impermeable to gases, stomatal pores are crucial in regulating water loss through transpiration and CO_2_ uptake for photosynthesis, accounting for approximately 95% of all gaseous fluxes through plants in terrestrial systems [3], with considerable implications for global hydrological and carbon cycles [4,5]. Stomata continually adjust aperture in response to fluctuations in environmental stimuli in order to balance photosynthetic CO_2_ demands with water loss for evaporative cooling [6,7]. Greater stomatal conductance has been correlated with crop yield in wheat, as it removes diffusional constraints of CO_2_ into the leaf for photosynthesis *and* enhances evaporative leaf cooling that maintains suitable temperatures for physiological processes [8]. Additionally, changes in climatic conditions can alter stomatal development and patterning [9]. Understanding stomatal anatomy and function due to changing environmental conditions is thus essential to evaluate the impact of climate change on plant growth, productivity and crop yield.

In order to function efficiently plants must integrate changing environmental signals in a hierarchical manner, adjusting stomatal behaviour to balance the ratio of CO_2_ fixed relative to water transpired, known as water-use efficiency (WUE). Stomata adjust aperture via changes in GC turgor in response to both internal and external cues [10]. Generally, stomata open with increasing light, low CO_2_ concentration [CO_2_], high temperature and low vapour pressure deficit (VPD) [11–15], with stomatal closure initiated by the opposite conditions. At any given moment in time, stomata must respond to combinations of these factors and the hierarchy of these responses is species-specific [16].

WUE is used as a critical measure of a plant’s ability to maintain photosynthesis and water status across a range of conditions and is often measured as the ratio of plant biomass to water lost by transpiration. Intrinsic WUE (*Wi*) (measured as the photosynthetic rate (*A*) divided by stomatal conductance (*g*_s_); [10,17,18]) provides a physiological assessment of the trade-off between photosynthetic carbon gain and the control of water loss by stomatal behaviour. High *g*_s_ enables high CO_2_ assimilation (*A*) but also leads to greater water loss [18–22]. Conversely, low *g*_s_ restricts CO_2_ uptake and negatively impacts on *A* [23,24] and biomass accumulation as a consequence [8]. A strong correlation between *g*_s_ and *A* has been demonstrated by many studies [20,23,25–28], however, this relationship, although conserved, is not always constant [29]. Under dynamic conditions changes in *g*_s_ can be an order of magnitude slower than adjustments in *A*, which leads to a disconnect between the two parameters [18,21,30,31], impacting both *W_i_* and assimilation rate in dynamic environments such as the field [18]. The magnitude and rapidity of these kinetic responses are also influenced by changing climatic conditions [16] and have gained increasing attention as a potential target for manipulation to improve both *A* and *g*_s_ [18,20,21,32]. Dumbbell-shaped GCs (which include many C4 grasses/crops) are generally associated with faster stomatal responses compared to C3 kidney-shaped GCs [2,31,33,34], a characteristic that may well confer significant advantages during more extreme temperature fluctuations of future climates. More rapid adjustments in aperture to dynamic environmental conditions enable plants to optimize water use, leaf temperature and photosynthetic carbon gain, which can buffer longer term changes in temperature.

Stomatal conductance is determined by anatomical features and functionality, both of which are species-specific and influenced by internal signals and environmental cues. Substantial work on stomatal patterning has elucidated many of the genes and regulatory pathways involved in stomatal development (stomatal density (SD) and size) and the impact of the environment on these. The mechanisms of osmoregulation for GC turgor changes are also well established, although further work is needed to fully understand all components of the signal transduction pathways that trigger movement. Several studies have manipulated genes within both pathways to alter SD and stomatal behaviour, with both positive and negative consequences for leaf physiology, illustrating the potential of genetic manipulation for crop improvements (see §2 below).

**Figure 1.**
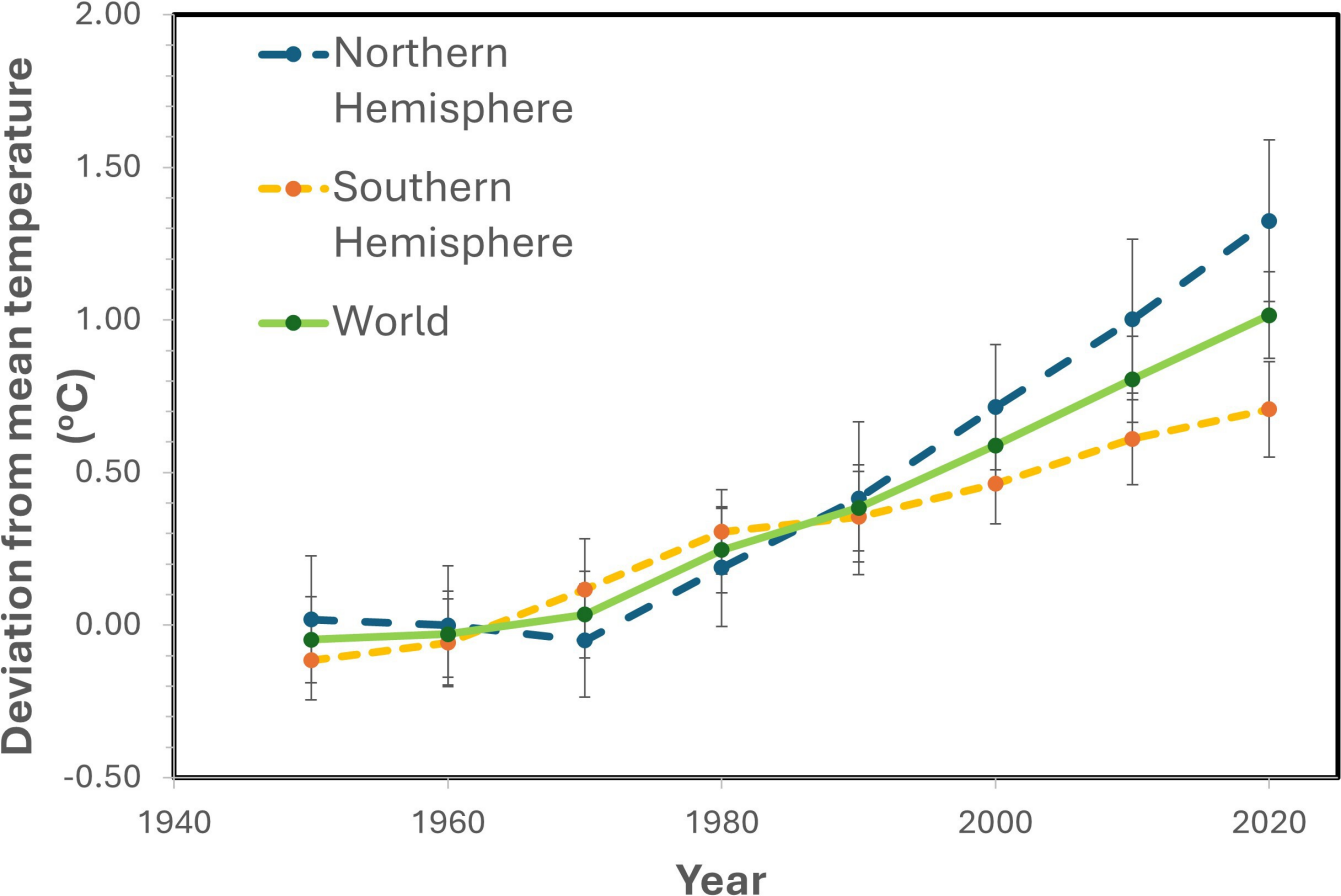
Global warming: comparison of combined land-surface air and sea-surface water temperature anomalies (mean ± s.d. for each decade between 1950 and present given as the deviation from the 1951−1980 mean) of Northern and Southern Hemispheres and World (data were obtained from https://ourworldindata.org/).

Climate change is predicted to result in a global temperature rise of 2.6–4.8°C by the end of the century [35], which, along with an increasing frequency of extreme temperature episodes [36], will have severe impacts on large areas of terrestrial ecosystems [37]. The frequency of these events will differ depending on geographical location, with more frequent episodes in the Northern Hemisphere (figure 1), which includes key agricultural land. Global warming is likely to influence stomatal behaviour directly as well as indirectly, via temperature-mediated changes in assimilation rate, transpiration, VPD and plant water status [38], while stomatal anatomical features, such as SD, are also impacted directly by heat during stomatal development [39–41]. Given the link between stomatal conductance, transpiration and photosynthesis/yield (see Introduction), any heat-induced change in *g*_s_, caused by alterations in SD and/or behaviour, has significant implications for crop productivity in future climates [42].

Here, we review current knowledge of stomatal anatomical and behavioural responses to temperature-driven changes, focusing on both rising temperatures and extreme heat events. Potential genetic targets for manipulation of relevant stomatal characteristics are discussed to illustrate the possible positive and negative impacts on photosynthesis, plant productivity and crop yield.

## 2. Impact of elevated temperature on genetic control of stomatal development and anatomical features

**Figure 2.**
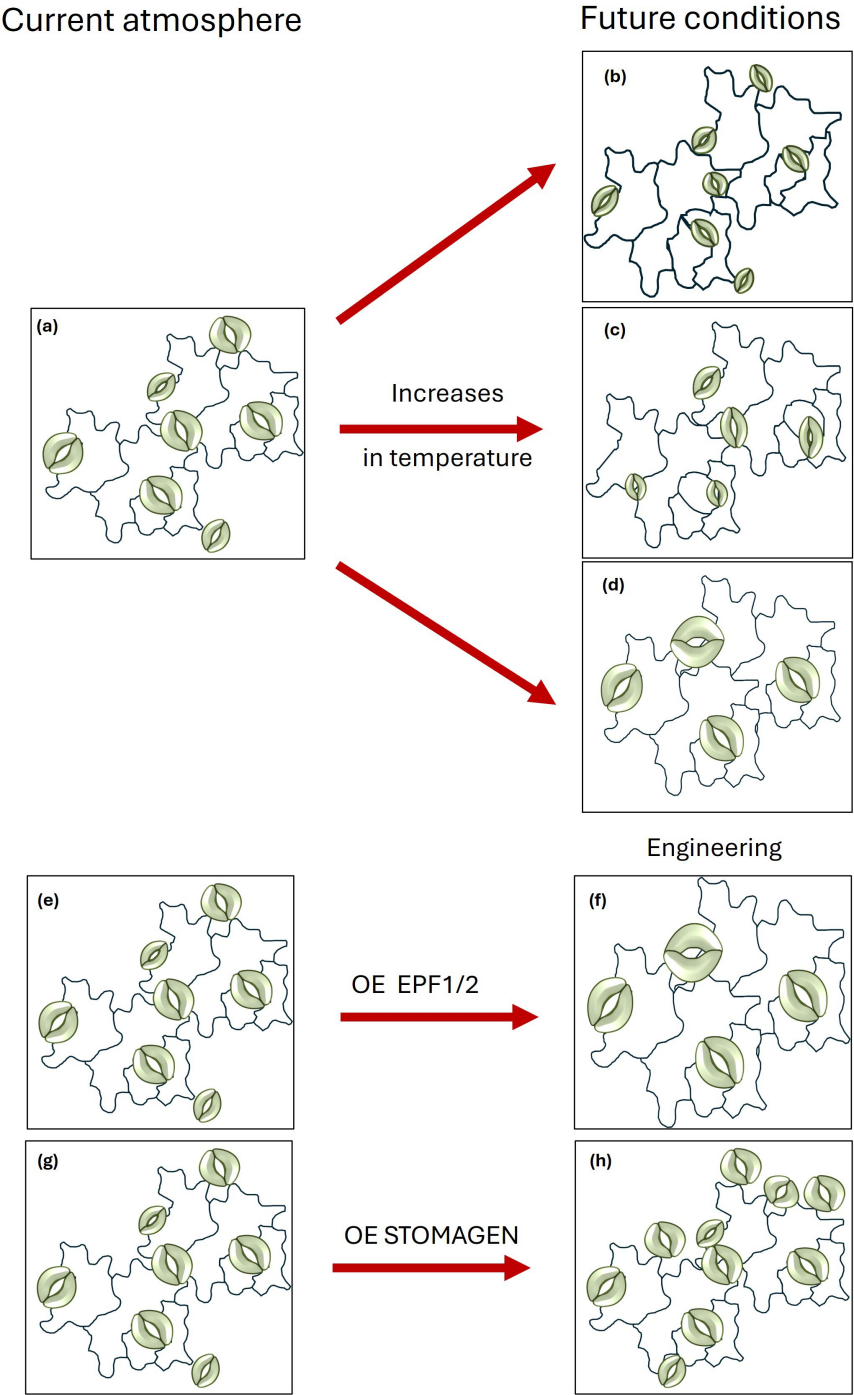
An illustration of the effect of elevated temperature on stomatal patterning, density (SD) and size (SS). (a) Current atmospheric, (b–d) possible future scenarios at higher temperatures that alter SD and SS: (b) no change in SD and reduced SS; (c) reduced SD and SS; (d) reduced SD and increased SS. Engineering using overexpression (OE) of EPF1/2 leads to reduced SD and typically greater SS (e,f), while OE of STOMAGEN increases SD and typically reduces SS but can also lead to stomatal clustering (g,h).

Stomatal patterning and SD are under genetic control, involving the signal peptides EPIDERMAL PATTERNING FACTORS 1 and 2 (EPF1/2) and STOMAGEN (EPFL9), which interact with receptor components TooManyMouths (TMM) and receptor kinases ERECTA (ER) or ERECTA-LIKE (ERL1 and 2), to activate mitogen-activated protein (MAP) kinases (e.g. YODA MAPKKK), which in turn inhibits transcription factors such as SPEECH-LESS (SPCH), MUTE and FAMA [43]. EPF1 and 2 activate MAP, decreasing SPCH and SD, whereas STOMAGEN has the opposite effect on the signalling cascade resulting in the development of a greater number of stomata on leaf surfaces [44–46]. Several studies have recently demonstrated the influence of heat on the genetic control of stomatal development [39–41]. For example, HEAT SHOCK PROTEINS 90 (HSP90s) convey heat-stress signals via the YODA cascade causing SPCH to break down and resulting in decreased stomatal densities ([47]; figure 2). Lau *et al*. [41] also highlighted the role of the PIF4 transcription factor, which accumulates at high temperatures and suppresses the expression of SPCH with similar reductions in SD. The PIF gene family is commonly associated with the repression of light signalling and PIF4 in particular has been linked to the auxin pathway [48], increasing transcription of the auxin-responsive gene IAA29 at high temperatures, which is involved in cell elongation and shade-avoidance responses. PIF4 may thus play an important role in coordinating stomatal development and density in response to both temperature and light [49], emphasizing the importance of considering combined effects of environmental factors on stomatal characteristics. Given the dual role of stomata in both CO_2_ uptake and water loss through transpiration, such combined responses are essential in balancing the often-conflicting demands experienced by the plant to maintain photosynthetic rates while limiting water loss to sustain leaf temperature via evaporative cooling. Li *et al*. [39] similarly showed that a number of stomatal lineage genes (SPCH, SCRM, FAMA, EPF1 and YODA) were tightly regulated by both light intensity and temperature in *Camellia sinensis* cultivars, with heat stress generally leading to the repression of stomatal development, via lower expression levels of SPCH, SCRM and FAMA alongside higher expression levels of negative regulators EPF1 and YODA. These studies highlight, alongside others [50], a number of potential targets for genetic improvement of WUE in changing climatic conditions, conveying water conservation, however, at the expense of a higher leaf temperature that could lower *A*. Additionally, a recent study by Lang *et al*. [51] showed that while core genes involved in stomatal development are under strong evolutionary constraint, regulatory genes show allelic variation enabling phenotypic plasticity and local adaptation. This study highlights the potential to exploit such natural variation of crop germplasms identified by genome-wide association studies and quantitative trait loci analyses to provide potential natural alleles that adapt well to the environment and thus are promising breeding targets.

Reduced SD in response to high temperatures has also been reported in herbarium specimens of *Quercus robur* [52] and *Pinus* [53], as well as *Brassica oleracea capitata* [54], while, in contrast, no response was seen in *Brassica oleracea acephala,* the C4 species *Zea mays* [54,55] and *Panicum maximum* [56], and higher SD have been reported in bluebunch wheatgrass, Norway spruce, Scots pine and *Dodonaea viscosa* [57–59], as well as roses [60]. It is, however, difficult to conclude that the observed increases in SD in the latter studies were the direct result of heat, as authors investigated the impact of other environmental parameters, such as concentrations of ozone, CO_2_ or water availability, respectively, alongside higher temperatures [54,55,57]. While these combined effects clearly represent natural conditions and reflect likely climate change scenarios, the observed species-specific variations in SD may be due to these combinations rather than temperature alone.

Rising temperatures impact stomatal size (SS) as well as density [52,61,62], with similarly varied effects on SS reported for different plant species. For example, Kapadiya *et al*. [63] found smaller stomatal length in heat-tolerant wheat varieties, compared with heat-suspectible plants when grown at different temperatures. Decreases in stomal size with heat were also observed in four alpine *Kobresia* meadow species by Zhang *et al.* [64], as well as increases in *Lolium perenne* and maize [65,66] and no change in Douglas fir and *Dodonaea viscosa* [67,68]. Again, these studies often investigated temperature in combination with changes in other environmental factors, making causative conclusions for temperature alone difficult.

A close relationship between SD and SS has been reported for many species and under various conditions, with higher density often coinciding with smaller stomata and vice versa [2]. Wu *et al*. [69] found no change in SS in *S. superba* with elevated temperature, despite lower SD. In contrast, no effect of temperature on SD was reported in *S. rehderianum*, yet there was a significant decrease in SS [69]. On the other hand, both Li *et al*. [39] and Rodríguez *et al*. [54] demonstrated an increase in SS alongside a decrease in SD, which allowed plants grown at high temperatures to maintain the same *g*_s_ as those experiencing lower temperatures, demonstrating the potential trade-off [70] and the importance of considering functional changes in combination with anatomical features in response to heat stress to fully understand the impact on *g*_s_ [16].

## 3. Impact of elevated temperature on stomatal behaviour

**Figure 3.**
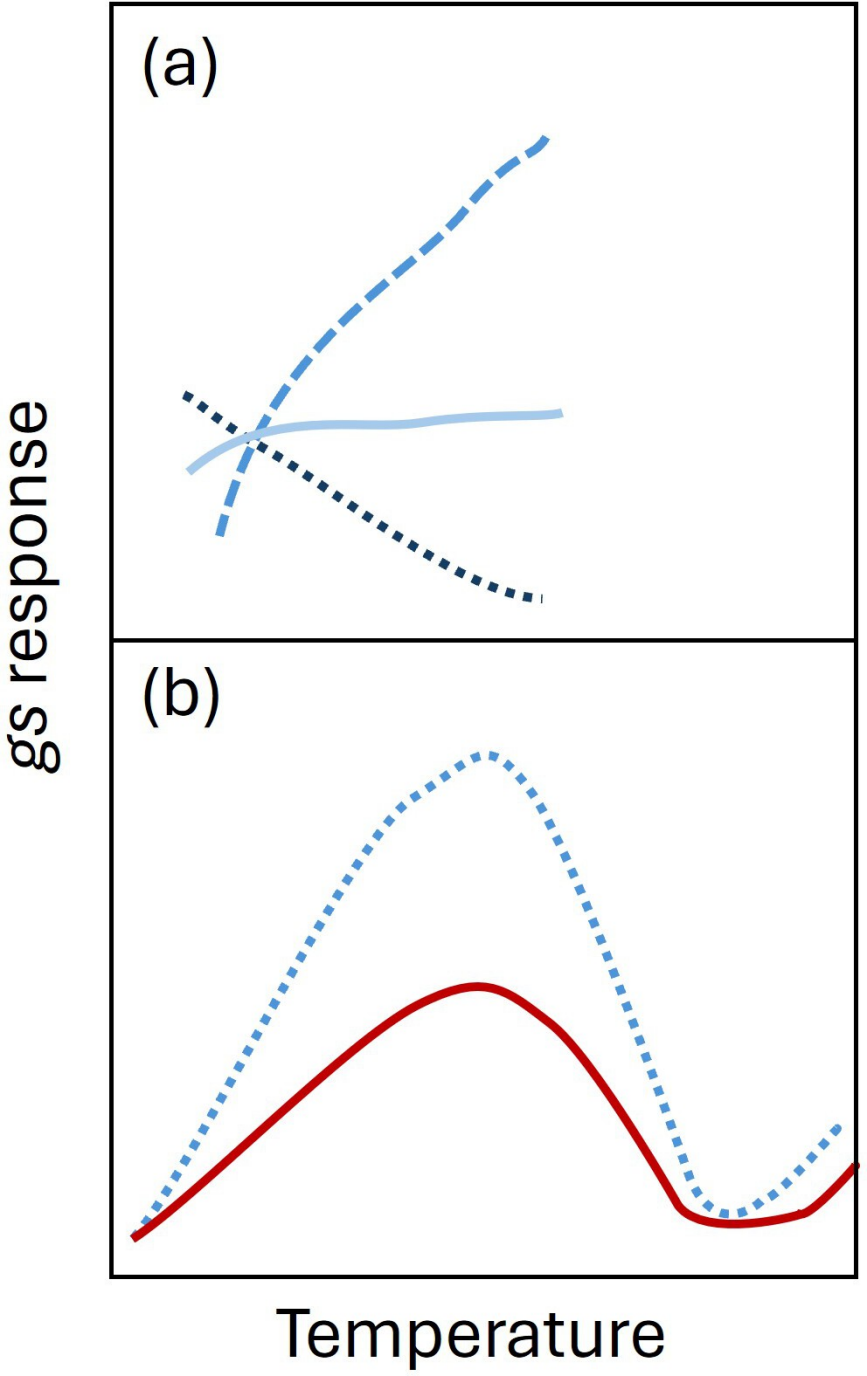
Species-specific *g*_s_ responses to increasing temperature. (a) Stomatal conductance has been reported to initially increase (mid-blue dashed line), decrease (dark blue dotted line) or not change (light blue solid line) with rising temperature. (b) Bimodal response in *g*_s_ has been shown in some species particularly with extreme temperatures (blue dotted line), and the magnitude of this response can be influenced by changes in other environmental factors such as elevated [CO_2_] (red solid line).

Even greater interspecific variation has been reported in the behavioural response of *g*_s_ to elevated temperatures [16,71]. Stomatal conductance may increase with rising temperature [38,72–74], decrease [75,76] or not respond directly at all [73,75,77], dependent on species and experimental protocol (growth conditions and experimental temperature ranges used). Across a large temperature range, a bimodal response may be seen [78], with *g*_s_ initially increasing, reaching a peak value before declining and then potentially rising again at higher temperatures [16], as plants attempt to balance CO_2_ uptake with evaporative cooling and water loss. These responses may be mediated by combined changes in environmental conditions. For example, elevated [CO_2_] may dampen the peaks in temperature responses as [CO_2_] is sufficient to maintain *A*, while drought/reduced water availability could reduce the initial response as water saving may become the main driver until lethal leaf temperatures are reached (figure 3).

Furthermore, interactions between environmental factors themselves must be considered to draw conclusions about direct effects. For instance, as leaf temperature rises so does VPD, which generally causes stomata to close [79] and most studies attribute this to a higher VPD, rather than heat itself. However, *g*_s_ did not decline with combined increases of temperature and VPD in a number of evergreen tree and herbaceous species, suggesting that a rise in temperature directly triggers stomatal opening [74]. Similarly, when VPD remained at 1 kPa and temperature was increased from 30 to 40°C, *g*_s_ was amplified by over 40% in both *Pinus* and *Populus* sp., showing a strong direct response of *g*_s_ to temperature in these species [38]. The same authors, and others [80], also demonstrated a decoupling of stomatal behaviour from mesophyll photosynthesis at high temperatures regardless of VPD, contradicting others who proposed that increases in *g*_s_ with rising temperature are inseparably linked to assimilation rates [81] and uncoupling occurred only due to transpiration-driven stomatal closing responses caused by a higher VPD at extreme temperatures [82]. However, any direct response of *g*_s_ to temperature and decoupling from photosynthesis warrants further consideration to inform models of stomatal control over photosynthesis [83–85], as these generally assume that the relationship between *A* and *g*_s_ is conserved over a wide range of environmental conditions, including temperature. In the same context, De Kauwe *et al*. [86] also pointed out the importance of such considerations to improve existing climate model projections. Recently, Mills *et al*. [87] have provided an excellent review of the direct response of stomata to temperature, which included the impact of various environmental factors on these responses and the natural species-specific variation.

Plants acclimate to their growth environments leading to species grown in similar thermal conditions exhibiting comparable patterns of *g*_s_ responses across a given temperature range, albeit at different magnitudes [88,89]. This may be due to both developmental alterations of stomatal patterning based on temperature altering SD (see §2 above), as well as behavioural adjustments impacting on stomatal opening. Optimal temperature ranges and tolerance to high temperatures are determined by the combination of all physiological adaptations and responses of a plant, including, e.g. kinetics of photosynthetic enzymes, which are species-specific [90,91],]. While stomatal opening with increasing temperature has been shown to occur regardless of photosynthetic biochemistry [92], given the different optimum growth temperatures of C3 (28–30°C) and C4 (26–35°C) plants as well as species-specific tolerance levels to high temperatures [93], it is likely that different magnitudes of *g*_s_ responses to increasing global temperatures are found when comparing these plant types [16].

**Figure 4.**
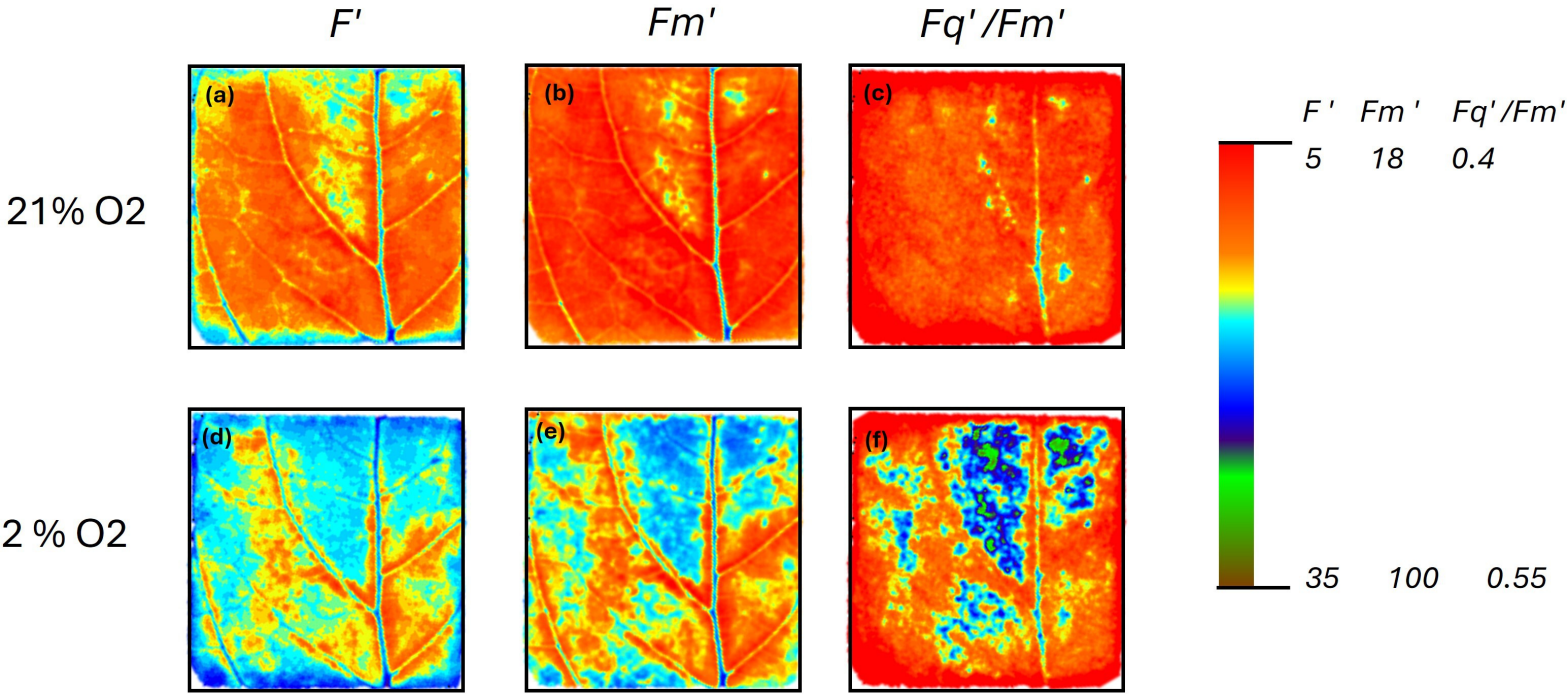
Non-uniform stomatal behaviour causes heterogeneous *g*_s_. The influence of stomatal behaviour can be visualized by comparing chlorophyll fluorescence imaging of photosynthetic efficiency *Fq′/Fm′* at 21% O_2_ (a–c)and 2% O_2_ concentrations (d–f).Images of steady state chlorophyll fluorescence, *F*′ (a,d) at 750 µmol m^−2^ s^−1^ PPFD, and maximum chlorophyll fluorescence in the light; *Fm*′ taken following a saturating pulse (b,e) were used to build images of the operating efficiency of PSII; *Fq*′ /*Fm*′ using the equation *Fm*′* − F*′/*Fm*′ (c,f). At 2% O_2_, photorespiration is removed, and therefore the chlorophyll fluorescence image of photosynthetic efficiency (*Fq*′/*Fm′*) illustrates patterns of use of the end products of electron transport for photosynthetic CO_2_ fixation, which reflects where stomata are open to allow CO_2_ to enter. Areas with smaller stomatal apertures (at 2% O_2_) have a lower *Fq*′/*Fm*′ as there is a reduced sink capacity for end products of electron transport as there is no O_2_ for photorespiration and limited CO_2_, demonstrating the influence of *g*_s_ on photorespiration that was clearly increased in the blue area.

Data using model simulations in conjunction with experimental evidence indicated that stomatal behaviour of C3 plants is more strongly influenced by temperature than C4 plants [94]. Additionally, stomatal closure impacts more severely on assimilation rates in C3 plants, as the carbon concentrating mechanism associated with C4 plants enables them to maintain higher *A* when *g*_s_ is lower. Crafts-Brandner *et al*. [95] demonstrated that *A* was inhibited at leaf temperatures above 38°C in C4 maize, however, transpiration rate increased with leaf temperature, indicating that inhibition was biochemical and not associated with stomatal closure. When exposed to combinations of soil moisture statuses and temperature ranges, a reduction in *g*_s_ in waterlogged soils occurred in maize and sorghum, without temperature dependence, while in millet and rice changes in *g*_s_ were strongly associated with temperature variations [96], showing again clear species-specific differences in responses. Any temperature-induced stomatal closure is also likely to increase photorespiratory losses in C3 plants, as a lower *g*_s_ restricts CO_2_ availability for photosynthesis [97,98], exposing Rubisco to relatively higher concentrations of O_2_, which together with an increase in leaf temperature further stimulates photorespiration [99]. Furthermore, changes in temperature alter VPD and plant water status, which can induce patchy stomatal behaviour [100]. This spatial heterogeneity in stomatal aperture over the leaf surface results in small areas of the leaf having distinctly different (‘patchy’) *g*_s_ values. The impact of patchy behaviour on carbon fixation and photorespiration can be visualized by comparing chlorophyll fluorescence images of photosynthetic efficiency (*Fq′/Fm′*) at 2 and 21% O_2_ concentrations [101]. Low O_2_ removes photorespiration, and therefore enables spatial photosynthetic efficiency to be imaged. Photosynthetic efficiency is high when stomatal apertures are also high and CO_2_ fixation uses the end products of electron transport. The uniformity observed in these areas at 21% O_2_ shows where photorespiration was taking place and illustrates the impact of *g*_s_ on these processes (figure 4).

## 4. Genetic control of temperature-dependent stomatal responses at extreme temperatures

With changing climates plants may experience episodic extreme temperatures that are likely to become more frequent in the future [102], with leaf temperature increases of 10–15°C above ambient resulting in severe heat stress, negatively impacting growth [103] (figure 1). Rice plants over-expressing EPF1 with reduced SD and SS were able to maintain photosynthesis at high temperature (40°C) and severe drought stress, due to conservation of water both before and during the drought event, enabling the mutants to increase *g*_s_ (via increased aperture) at 40°C to levels equivalent to the control lines [104]. Although the increased *g*_s_ incurred a reduction in *W_i_*, these plants showed greater survival under drought conditions, indicating that stomatal anatomical changes could be beneficial under water stress and extreme temperatures. Fine-tuning stomatal characteristics, therefore, holds great promise to produce crop plants that are able to survive and perform in future atmospheres [104].

While stomatal opening at high temperature has been well established in many plant species, the exact mechanism is less well understood. Using epidermal peels of *Arabidopsis*, Kostaki *et al*. [105] suggested that full stomatal opening at high temperature requires blue light and involves phototropin photoreceptors, with only partial reliance on their downstream target BLUE LIGHT SIGNALING1 (BLUS1; [106,107]), as well as plasma membrane (PM) H^+^-ATPase activity [105]. However, how and which of these signalling components are influenced by high temperature remains to be investigated. Pankasem *et al*. [80] used a host of *Arabidopsis* mutants to illustrate that impairment in the blue-light photoreceptors phot1−5/phot2-1 resulted in some reduction in stomatal opening in response to temperature. However, plants with mutations in CO_2_ sensing and signalling pathways including ht1, mpk12/mpk4-gc and cbc1/cbc2 entirely lacked a stomatal temperature response, suggesting a direct link to carbon assimilation through reduced internal CO_2_ from heat stimulated photosynthesis. The fact that the same outcome was observed in monocot *Brachypodium distachyon* mutants implies this to be a conserved mechanism across plant types. Interestingly, at higher temperatures the link with photosynthesis was uncoupled, as also suggested by others . GC osmoregulation for heat-inducted stomatal opening has been suggested to involve triacylglycerol synthesis and degradation (usually associated with the blue-light stomatal response), which provide the energy and carbon skeleton for stomatal opening [108]. In order to explain high temperature-induced stomatal opening in the dark, Devireddy *et al*. [109] presented evidence for a phototropin-independent pathway mediated by reactive oxygen species production, which altered stomatal aperture within minutes. Similarly, Liu *et al*. [103] investigated the control of H_2_O_2_-induced stomatal closure to identify possible genetic targets to improve heat tolerance in rice. In this study, authors assessed the survival rates following heat stress as a measure of heat tolerance and concluded that mutant plants of the chloroplastic MDHAR (OsMDHAR4) gene had improved heat tolerance via increased stomatal closure and reduced water loss caused by H_2_O_2_ accumulation, thus concluding that the OsMDHARA4 gene negatively impacts on heat resistance. A more recent study has explored the cell mechanisms involved in the complex and opposing stomatal responses to combined heat and drought stress [110]. This study revealed a signalling pathway integrating both opening and closing responses related to high temperature and drought, via the heat activation of TARGET OF TEMPERATURE 3 (TOT3), which controls the activity of plasma membrane H^+^-ATPases for stomatal opening, and OPEN STOMATA 1, involved in stomatal closure inactivating TOT3 by phosphorylation.

## 5. Future perspective and engineering guard cells and stomata

A greater understanding of the underlying genetic mechanisms that govern stomatal development coupled with advancements in genetic engineering of stomatal function provides new opportunities to develop crops with greater photosynthesis, *W_i_* and bolstered resistance to climate-change induced stresses, such as high temperature alone as well as in combination with other factors. This is only possible with the advancements of new tools to engineer crops using tissue-specific promoters that restrict expression to cell/tissue/organ type, such as the *Solanum tuberosum* KST1 GC-specific promoter [111,112]. Inducible promoters, such as the β-estradiol vector, can be specifically induced by different environmental cues and are also useful tools for expression under desired conditions or time [113,114]. Recent advancement in promoter editing mediated by CRISRP-Cas9 could also provide new tools for fine-tuning expression levels of desired genes [115], circumventing the need for transgenics that is still under strict regulation in many countries.

Using our knowledge of the impacts of climate change on stomatal patterning and behaviour, in conjunction with such innovative tools could allow us to future-proof crop performance in the face of climate change and global warming specifically.

As stomatal conductance is determined by several stomatal anatomical characteristics as well as functional responses there is a plethora of opportunities to tailor stomatal physiology to create the desired crop ideotype for particular agricultural locations, including those more likely to experience increased warming and heat events (figure 1). Stomatal morphology can be manipulated to control *g*_s_ in several ways, including SD, SS and GC shape, as well as by manipulating the epidermal cells that surround them. Manipulating SD has proven to be extremely useful to alter the delicate balance between carbon gain and water loss, which is particularly relevant under high temperature-induced drought conditions. Genetic manipulations decreasing SD have been demonstrated to improve *W_i_* as reviewed recently by Bertolino *et al*. [50]. Reduced SD has been achieved through the overexpression of the epidermal patterning factor EPF1 in a variety of plant species, including *Arabidopsis*, rice, wheat, barley and poplar [44,104,116–119]. Similarly, overexpression of EPF2 caused up to a 42% reduction in SD in C4 sorghum, resulting in lower *g*_s_ and greater *W_i_*, with no effect on *A* [120]. On the other hand, Lunn *et al*. [121] reported no change in *g*_s_ or *W_i_* in EPF2 overexpressing sugarcane despite up to a 38% reduction in SD in these plants, suggesting an increase in stomatal aperture compensating for lower stomatal numbers. Additionally, overexpression of STOMATAL DENSITY and DISTRIBUTION1 (SSD1) reduced SD, *g*_s_, and therefore water consumption in maize and tomato [43,122]. The reduction in SD using these approaches could provide strategies for reducing water consumption during episodes of high evaporative demand driven by higher temperatures. However, this could lead to lethal leaf temperatures that would negatively impact plant performance.

Increasing SD may be useful when aiming to improve crop yields where temperatures are increasing but water scarcity is not a factor to facilitate greater evaporative cooling via transpiration to maintain adequate leaf temperature. Tanaka *et al*. [123] investigated the effects of overexpressing STOMAGEN/EPFL9 in *Arabidopsis* and found that SD was increased by 372%, GCs had significantly reduced cell length and stomatal index was also increased compared to wild type (WT). Transpiration in ST-OX plants was increased by 82%, however, the decrease in *W_i_* was not significant. Interestingly though, the relatively large 30% increase in *A* did not translate into higher leaf area or whole plant biomass.

Recent work has shown that manipulating EPF1/2 differentially impacts SD on abaxial and adaxial leaf surfaces, with greater increases in SD observed on the abaxial surface in EPF1/2 mutants [124]. Since the adaxial surface has previously been shown to contribute up to 50% of *g*_s_ and C assimilation in wheat [125], elucidating the mechanisms that control the development of stomata on the two surfaces could provide a more targeted approach to manipulate SD on different leaf surfaces with a view to improving thermal tolerance and photosynthesis in future climates.

As ultimately operational *g*_s_ is determined by the aperture of the pore, manipulating GC metabolism, osmoregulation and/or signalling pathways represents another attractive target for stomatal engineering (using GC-specific promoters; recently reviewed by Lemonnier & Lawson [126]). Manipulating solute transport in particular, by changing the number of transporters, could be used to adjust the rate of ion influx or efflux and osmoregulation in the GCs thereby influencing both overall aperture and therefore *g*_s_ as well as possible rapidity of responses, which is particularly important in dynamically changing environments (e.g. rapid temperature fluctuations). Wang *et al*. [127] demonstrated the potential of this approach by overexpressing H^+^-ATPase (AHA2) in *Arabidopsis* under the control of the GC-specific promoter GC1 [128] and reported enhanced stomatal opening that lead to an increase in photosynthesis and plant growth [127]. This strategy could also be used to enhance evaporative cooling. GC-specific downregulation of SUCROSE TRANSPORTER 1 (SUT1) resulted in lower *g*_s_ and improved *W_i_* [129], though in conjunction with a decrease in net *A*, which resulted in reduced growth, although the lower water consumption improved tolerance to water deficit that could provide a route to reducing water loss under high temperatures. Overexpression of a synthetic light-gated K^+^channel BLINK1 in *Arabidopsis* GCs enhanced stomatal kinetics, improving the rate of stomatal opening and closing in response to light changes [130], which often coincide with fluctuations in temperature. The accelerated stomatal kinetics provided by BLINK1 facilitated enhanced carbon assimilation during periods of high light while conserving water when light availability was lower and establishes the potential of increasing the rapidity of stomatal kinetics as a target for improving both *A* and *W_i_* [10,18,20,21,31].

Improving stomatal kinetics can also be achieved by altering GC sensitivity to intracellular signals. The most well-studied example of this is the dual-function sugar sensing and phosphorylating enzyme hexokinase [131]. When overexpressed in the GCs, transgenic plants exhibited accelerated stomatal closure, reduced *g*_s_ and transpiration while the rate of photosynthesis was maintained, increasing *W_i_* [132–135]. Transgenic tomato and *Arabidopsis* plants overexpressing hexokinase in their GCs exhibited reduced transpiration and 20% higher WUE under normal conditions compared with WT, while maintaining comparable rates of *A* [136]. Co-expression of GC-specific hexokinase together with the tuberization SELF-PRUNING 6A gene (SP6A; under the expression of a leaf/stem-specific StLS1 promoter) in potato [135] resulted in smaller, shorter stomata, leading to approximately 30% lower transpiration with limited effect on *A*. This led to an approximately 30% increase in WUE in the transgenic lines and a 30–70% higher tuber yield (despite green biomass being lower). Moreover, the tuber yield remained high even under drought and heat stress conditions, while in wild-type plants yield was reduced by approximately 70%, demonstrating the potential of manipulating stomatal processes to future-proof crops in the face of global warming.

## Data Availability

This article has no additional data.
